# The first complete mitochondrial genome and phylogenetic analysis of deep-sea asteroid, *Leptychaster arcticus* (Valvatacea: Paxillosida: Astropectinidae)

**DOI:** 10.1080/23802359.2024.2404208

**Published:** 2024-09-23

**Authors:** Philjae Kim, Chang Rak Jo, Young Sun Song, Jung-Hye Won

**Affiliations:** a National Marine Biodiversity Institute of Korea, Seocheon-gun, Chungcheongnam-do, Korea; b National Institute of Fisheries Science, Pohang, Korea

**Keywords:** *L. arcticus*, deep sea, Asteroidea, mitogenome, molecular phylogenetic relationship

## Abstract

The complete mitochondrial genome of *Leptychaster arcticus*, deep-sea inhabited asteroid, was examined in this study. The complete mitogenome of *L. arcticus* is 16,253 bp in length and contains 13 protein-coding genes, 22 transfer RNA genes, and two ribosomal RNA genes. No gene rearrangements or deletions were observed in compared to other Paxillosida. The ND4L and ND3 genes have ‘ATT’ as its start codon, which is a feature that has been found in previous echinoderm mitochondrial studies. In the ML tree analysis based on the superorder Valvatacea, it was difficult to establish the molecular phylogenetic relationship at lower taxonomic levels, such as order and family, due to the lack of asteroid molecular data available. Therefore, we expect to contribute to the expansion of the data and determine the phylogenetic positioning in future studies.

## Introduction

*Leptychaster arcticus* (M. Sars, 1851) inhabits the deep sea with collection records from 560–2,400 m depth (Sladen 1889; Bell 1892; Ringvold & Andersen 2016). The organisms that live in the deep-sea, such as some echinoderms, are mostly difficult to study using conventional survey methods such as SCUBA diving or netting. Therefore, those taxa groups may not have been as actively studied as their shallow-water dwelling relatives. Currently, among the 11 *Leptychaster* species, none of the mitochondrial complete genome had been published in NCBI, and the only three species were analyzed partial genes, such as H3, 12S, 16S and COI (Mah and Foltz 2011). In particular, no molecular data were available for *L. arcticus*. Here, we aim to examine the mitochondrial genome characteristics of *L. arcticus* completely in the first within their genus, and expect to expand the molecular reference data of deep-sea echinoderms.

## Materials and methods

**Figure 1.**
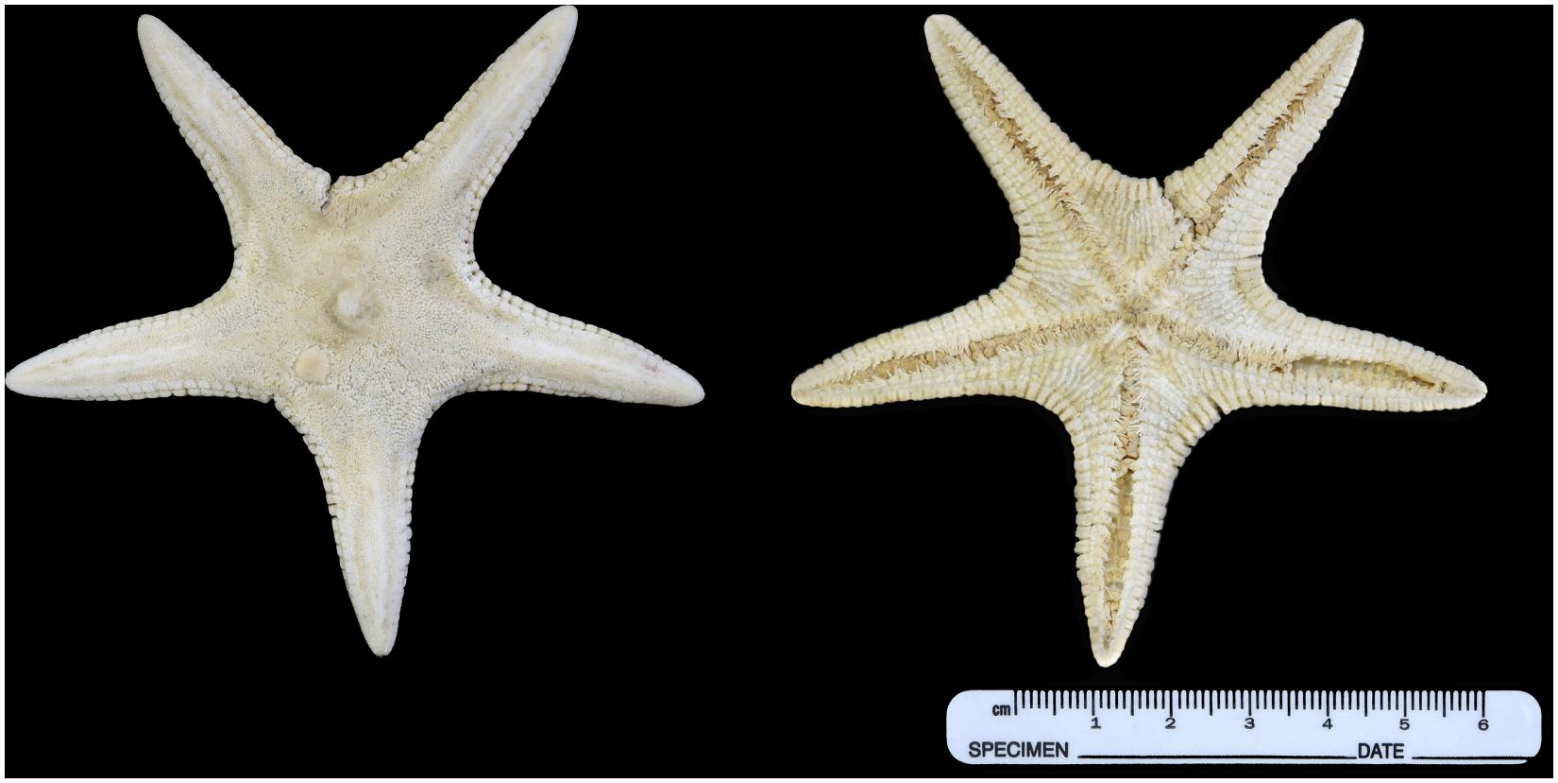
*Leptychaster arcticus* (M. Sars, 1851). Aboral (left) and oral (right) view within alcohol preserved. This photograph was taken by authors.

The specimen of *L. arcticus* (voucher code: MABIK IV00173150) analyzed in this study was collected by trawling at a depth of 300 m from the East Sea (37°00′48.68″N, 129°43′00.16″E) on 5 November 2022. For identification, the morphological characteristics were examined according to Sladen (1889) and Shin and Rho (1996). The organism was immediately frozen and transported to the laboratory. For mitochondrial DNA isolation, tube feet were used from the frozen specimen. Then, it was fixed in absolute alcohol for specimen preservation, and photographs were taken with Nikon D810A (Figure 1). A voucher specimen was stored at the National Marine Biodiversity Institute of Korea under the voucher number MABIK IV00173150 (Seocheon, Korea) (https://www.mbris.kr/, Philjae Kim, philjaek@mabik.re.kr, Seochen, Korea). First, mitochondria were isolated from tube feet minced on ice using the Qproteome Mitochondria Isolation Kit (QIAGEN, Hilden, Germany). In addition, mt-DNA extracted using DNeasy Blood & Tissue DNA Isolation Kit (QIAGEN) and stored in deep freezer until use. We then prepared the PCR product with mt-DNA using REPLI-g Mitochondrial DNA Kit (QIAGEN) for NGS analysis, and NGS library was prepared using QIAseq FX Single Cell DNA Library Kit (QIAGEN). The Illumina HiSeq 4000 platform (San Diego, CA, USA) was used for sequencing at GnC Bio Co. (Daejeon, South Korea). Raw data treatment step that including trimming, contig assembly, and annotation was performed using Geneious Prime (v. 2023.1.1) (Figure S1). Gene annotation was performed using MITOS2 web and tRNAscan-SE 2.0 (Bernt et al. 2013; Chan and Lowe 2019). The dataset consisted of 13 PCGs of the total of 44 asteroid mitochondrial genomes, consisted of 41 Valvatacea and three Forcipulatacea. As outgroups, we used three Forcipulatacea species. The maximum likelihood (ML) tree method was used, and it was performed with 1,000 replicates using PhyML 3.1, and the best fit model, GTR + I + G, was estimated by jModel Test (Guindon and Gascuel 2003; Guindon et al. 2010; Darriba et al. 2012).

## Results

**Figure 2.**
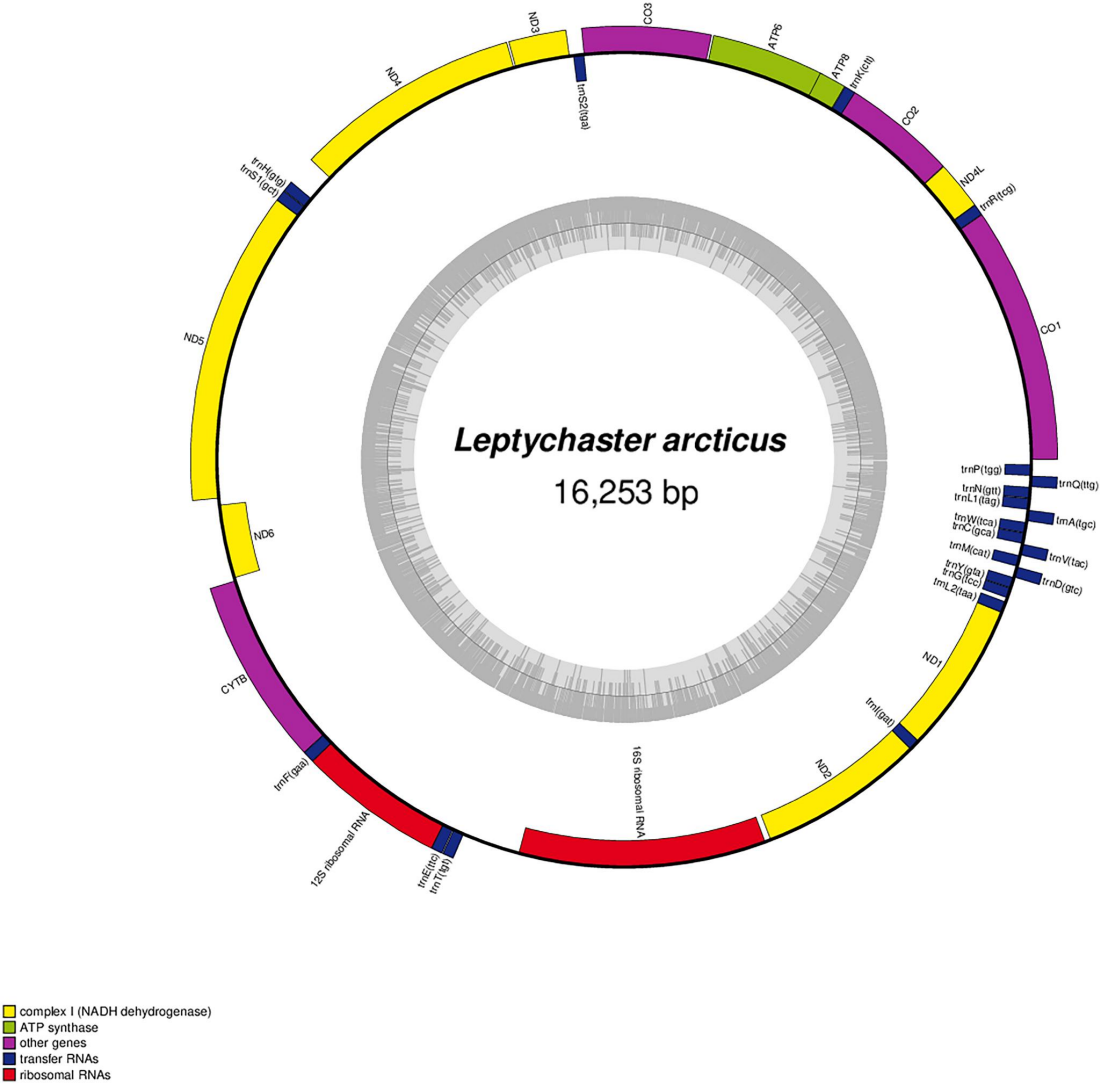
Circular map of *Leptychaster arcticus* mitochondrial complete genome with gene annotation and direction shown on the map. The inner and outer color bars on circle indicate the gene direction reverse and forward, in respectively.

The complete mitochondrial genome of *L. arcticus* (OR142187) was 16,253 bp in length and consisted of 37 genes, including 13 protein-coding genes (PCGs), 22 transfer RNAs and two ribosomal RNAs (Figure 2). Furthermore, in supplementary Figure 1, the coverage depth was consistent and sufficient in the entire assembled region, confirming the reliability of the assembled sequence. The mitochondrial gene rearrangement occurs in some echinoderms, and this is considered as a new paradigm for determining the specific phylogenetic relationships and evolutionary events of them (Smith et al. 1989, 1993; Perseke et al. 2008; Galaska et al. 2019). However, no gene rearrangement or deletion occurred in this species, and the gene order was identical to that of four species (AB183558, MH648613, MZ702701, OP289522), which belongs to the same order Paxillosida. Furthermore, there was no gene rearrangement observed in Valvatida. In terms of nucleotide base composition, it was found to have the lowest G content, and the total content was as follows: 32.6% A, 30.0% T, 23.9% C and 13.5% G. Only ND2 has an initiation codon with ‘GTG’, and 10 of the remaining PCGs started with ‘ATG’ except ND4L and ND3. ND4L and ND3 were started with ‘ATT’. A previous study of the mitochondrial genetic code in echinoderms suggested that ‘ATA’, ‘ATC’ and ‘ATT’ could be considered as start codon despite their reservations (Cantatore et al. 1989). ‘ATT’ is the most dominant annotated start codon in ND3 among the invertebrates including echinoderm (Donath et al. 2019). It was also suggested that two genes, ND3 and ND4L, are initiated with ‘ATT’ or ‘ATC’ according to published mitogenomes assembly of asteroid species (Quek et al. 2021). Meanwhile, the CytB has an incomplete terminal codon ‘T ––’. The 10 PCGs (COI, ND4L, COX2, ATP8, COX3, ND3, ND4, ND6, ND2, ND1) and two PCGs (ATP6, ND5) have ‘TAA’ and ‘TAG’ as terminal codon, respectively.

## Discussion and conclusion

**Figure 3.**
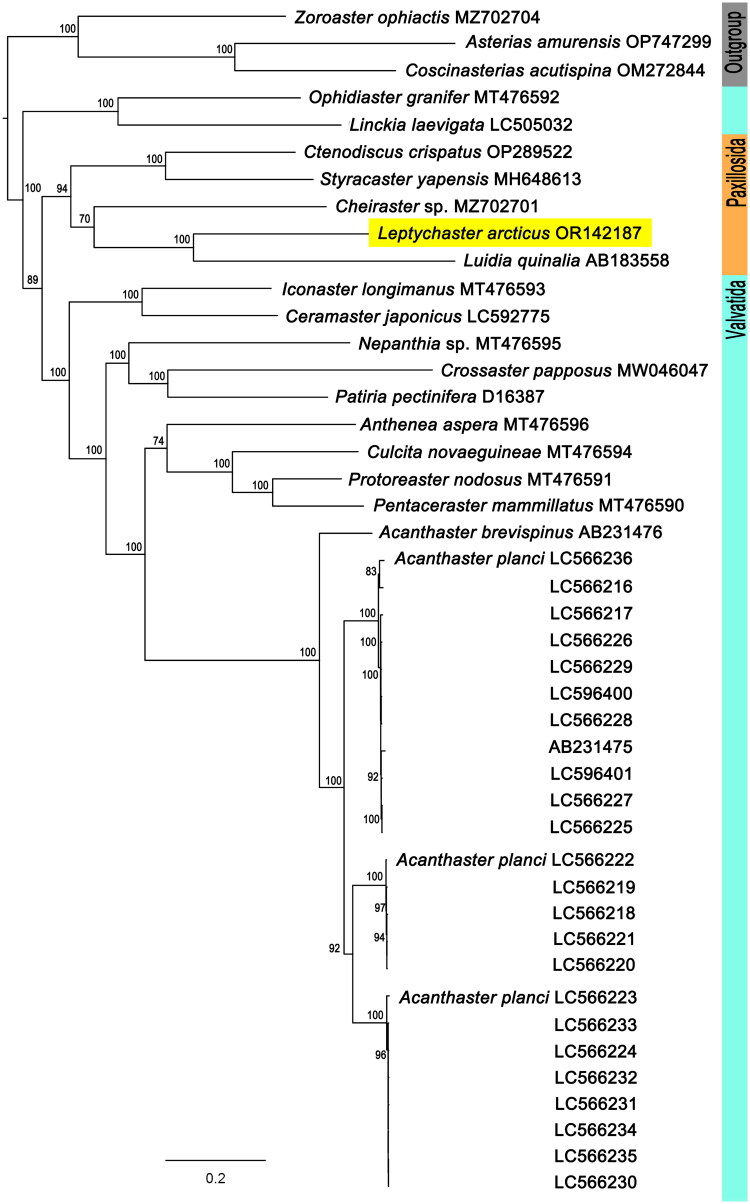
The molecular phylogenetic position of *Leptychaster arcticus*. The maximum likelihood tree was constructed with GTR + I + G and 1,000 bootstrap replicates based on 13 PCG sequences 44 asteroid species, including *L. arcticus* (OR142187). The three Forcipulatacea species (zoroasteridae, MZ702704; asteriidae, OP747299, OM272844) were used as outgroup. Bootstrap support values are indicated on each node as ≥70. The following sequences were used: AB183558 (Matsubara et al. 2005), AB231475, AB231476 (Yasuda et al. 2006), D16387 (Hyouta et al. 1987), LC505032 (hiruta et al. 2005), LC592775 (Yamamoto et al. 2021), MH648613 (Mu et al. 2019), MT476590–MT476596 (Quek et al. 2021), MW046047 (Nam et al. 2021), MZ702701, MZ702704 (Sun et al. 2022), OM272844 (Han et al. 2022), LC566216–LC566236, LC596400, LC596401 (Yuasa et al. 2021).

We determined the characteristics of the complete mitochondrial genome and deposited it in GenBank of NCBI (http://www. ncbi.nlm.nih.gov). Also, we aim to determine the molecular phylogenetic position of L. arcticus, however, Paxillosida mitogenomes are inadequate to solve with it. Thus, we tried to including Genbank records at least one representative per family of Paxillosida. In accordance with the lack of public molecular data of asteroids in GenBank, phylogenetic analysis was conducted in higher taxonomic level. We prepared the dataset based on the complete mitochondrial genomes of Paxillosida and Valvatida published in GenBank. We compared Paxillosida (4 families; 5 species; 5 sequences) and Valvatida (6 families; 13 species; 36 sequences) belonging to Valvatacea, and Forcipulatacea (2 families; 3 species; 3 sequences) was selected as an outgroup following Mah and Foltz (2011). We used a total of 44 mitogenome data, including our data. According to our result, Paxillosida including *L. arcticus* was shown in monoclade, and they were placed in the Valvatida clade (Figure 3). However, it was difficult to determine whether they were polyphyletic or monophyletic taxa because of the number of sequences and species were not sufficient to resolve their molecular phylogenetic relationships. We have not been able to fully determined the taxonomic relationships in Valvatacea in this study. However, we expect that our results will contribute to the expansion of the molecular data on deep-sea inhabited asteroids and to determining the complete mitogenome-based molecular phylogenetic positioning in future studies.

## Supplementary Material

Supplemental Material

## Data Availability

The genome sequence data supporting the results of this study are openly available in GenBank of NCBI at [https://www.ncbi.nlm.nih.gov] (https://www.ncbi.nlm.nih.gov/) under the accession no. OR142187. The associated BioProject, SRA and Bio-Sample numbers are PRJNA973727, SRR24758789, and SAMN35124885 respectively.
