# Supplementary material for: The first complete mitochondrial genome and phylogenetic analysis of deep-sea asteroid, Leptychaster arcticus (Valvatacea: Paxillosida: Astropectinidae)
Source: Mitochondrial DNA B Resour. 2024 Sep 23;9(9):1263–7. doi: 10.1080/23802359.2024.2404208 (PMC11423521; doi:10.1080/23802359.2024.2404208)
Supplement: Supplemental Material [file TMDN_A_2404208_SM5126.docx]

Figure captions


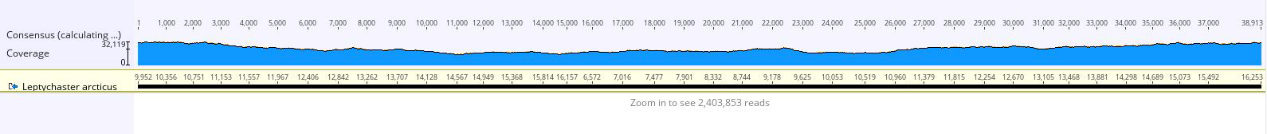


Figure S1. Overall coverage depth of the mitochondrial complete genome assembly of *Leptychaster arcticus*. The coverage depth means the number of times a randomly sequenced short nucleotide is read during assembly. The figure was generated using Map to reference assembly method of Geneious Prime. The coverage depth graph was provided as it was analyzed without managing. The blue graph indicates assembled mitogenome coverage depth.
